# *Arabidopsis* Voltage-Dependent Anion Channel 1 (AtVDAC1) Is Required for Female Development and Maintenance of Mitochondrial Functions Related to Energy-Transaction

**DOI:** 10.1371/journal.pone.0106941

**Published:** 2014-09-05

**Authors:** Xiaodi Pan, Ziwei Chen, Xueyong Yang, Guoqin Liu

**Affiliations:** State Key Laboratory of Plant Physiology and Biochemistry, College of Biological Sciences, China Agricultural University, Beijing, China; The University of Melbourne, Australia

## Abstract

The voltage-dependent anion channels (VDACs), prominently localized in the outer mitochondrial membrane, play important roles in the metabolite exchange, energy metabolism and mitochondria-mediated apoptosis process in mammalian cells. However, relatively little is known about the functions of VDACs in plants. To further investigate the function of AtVDAC1 in *Arabidopsis*, we analyzed a T-DNA insertion line for the *AtVDAC1* gene. The knock-out mutant *atvdac1* showed reduced seed set due to a large number of undeveloped ovules in siliques. Genetic analyses indicated that the mutation of *AtVDAC1* affected female fertility and belonged to a sporophytic mutation. Abnormal ovules in the process of female gametogenesis were observed using a confocal laser scanning microscope. Interestingly, both mitochondrial transmembrane potential (ΔΨ) and ATP synthesis rate were obviously reduced in the mitochondria isolated from *atvdac1* plants.

## Introduction

Voltage-dependent anion channels (VDACs) are known as the most abundant proteins in the outer mitochondrial membrane [1]–[3]. As eukaryotic porins across the outer mitochondrial membrane, VDACs mediate the exchange of metabolites such as ATP, NADH, and ions [4]–[7] between the mitochondria and the cytoplasm, which is very significant for mitochondrial functions and cellular energy transactions [8]. VDAC, known as a component of the permeability transition pore in mammalian cells, is involved in mitochondria-mediated apoptosis together with the adenine nucleotide translocator and other molecules [9]–[12].

VDACs are widely present in fungi, plants and animals. Two forms of *VDAC* genes were found in yeasts [13], and three isoforms (VDAC1, VDAC2 and VDAC3) in mammalian cells [14]. Mammalian VDAC members function in male reproduction, the synaptic plasticity, and normal glucose homeostasis [15]–[17]. In plants, VDAC isoforms were identified in many species, such as *Oryza sativa*, *Nicotiana tabacum*, *Lotus japonicus*, *Triticum aestivum*, *Solanum tuberosum*, *Arabidopsis thaliana* [7], [18]–[26] and other species (see review [27]). The information about structure, subcellular location, and physiological functions of plant VDACs has been explored gradually, although our knowledge of VDACs in plants is much less than that in animals. VDACs in plants display similar electrophysiological and topological properties as other eukaryotic VDACs [27]; however, apart from roles in the growth and development, plant VDACs have their own specificities such as functions in biotic and abiotic stress responses [28]. The *Arabidopsis thaliana* genome encodes five VDAC isoforms, and four of them have been cloned [22]. In the past several years, T-DNA knockout mutants were used to analyze the functions of *Arabidopsis* VDAC members. In 2011, Tateda and coworkers first identified T-DNA insertion mutants of *AtVDAC1* (At3g01280), *AtVDAC2* (At5g67500), *AtVDAC3* (At5g15090), *AtVDAC4* (At5g57490), and reported that *AtVDAC1*, *AtVDAC2* and *AtVDAC4* played important roles in plant growth, leaf and pollen development, as well as in maintaining the steady state of the mitochondrial membrane potential; in particular, *vdac1* plants produced slender and shorter siliques [29]. The number of pollen grains, the pollen germination rate, and the tube length of germinated pollen were dramatically reduced in the *vdac1* mutant [28]. In 2012, Robert and coworkers demonstrated that three AtVDACs were dual-localized in both plasma membrane and mitochondrial membrane, and some mutant phenotypes were related to mitochondrial functions. They suggested that the decreased seed set in the *atvdac1* mutant was not related to gametophytic sterility but rather followed from zygotic or early embryo lethality [30].

In the process of aerobic respiration, mitochondria are the pivotal organelles for ATP synthesis, through oxidative phosphorylation. Electron flow past the electron transport chain located in the inner mitochondrial membrane is coupled to proton translocation out of the matrix. The proton gradient across inner mitochondrial membrane drives ATP synthesis through oxidative phosphorylation, and the charge imbalance resulted from the generation of an electrochemical gradient across the inner mitochondrial membrane forms the basis of the inner mitochondrial transmembrane potential [31], [32]. Then what is the function of the abundant protein, VDAC, across the outer mitochondrial membrane? In mammalian cells, VDAC is involved in the permeability transition pore which mediates mitochondrial permeability transition leading to the loss of mitochondrial transmembrane potential, and participates in mitochondria-mediated apoptosis [33]. Do VDACs have underlying roles in regulation of mitochondrial transmembrane potential and/or ATP synthesis in plants? In *Arabidopsis atvdac1* plants, what is responsible for reduced seed set? Is AtVDAC1 involved in male or female development? Furthermore, is AtVDAC1 involved in the processes of sporogenesis and gametogenesis before fertilization or the processes of zygogenesis and embryogenesis? Exploring these issues will help us to find out more information about functions of AtVDAC1.

In this study, we investigated the function of *AtVDAC1* using the T-DNA insertion mutant *atvdac1* in reproductive development. The phenotype in *atvdac1* plants, reduced seed set, results from plenty of undeveloped ovules in siliques. Results indicate that AtVDAC1 is required for female development and involved in maintaining the steady state of mitochondrial transmembrane potential and ATP synthesis. This result is significant for further understanding of functions of AtVDAC1 in the reproductive process.

## Results

### Mutation of *AtVDAC1* Causes Reduced Seed Set

**Figure 1:**
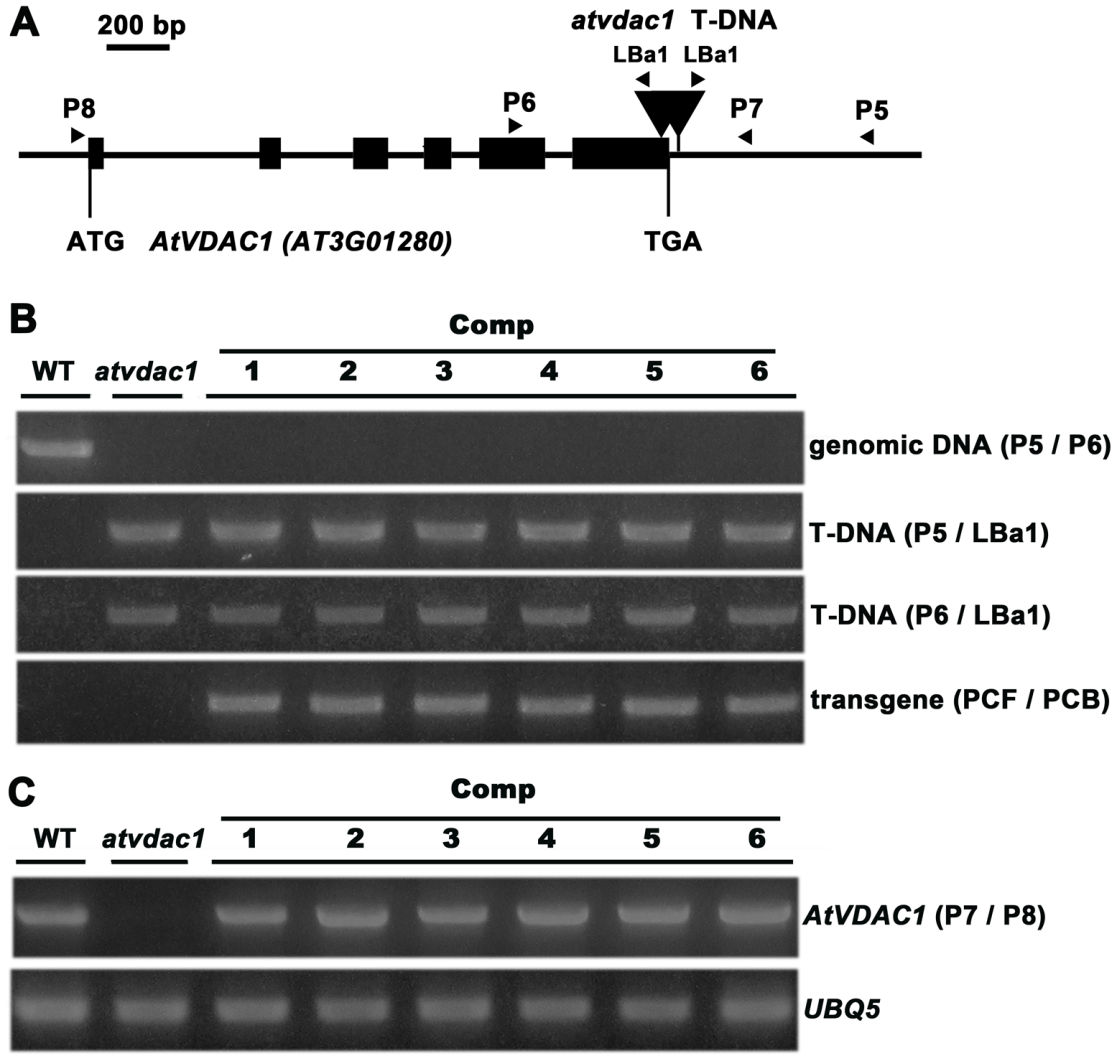
Molecular confirmation of isolated *atvdac1* mutant and its gene complementation. (A) Schematic diagram of the *AtVDAC1* gene structure and the T-DNA insertion sites in the *atvdac1* mutant. Closed boxes indicate exons, and arrowheads indicate the positions of primers used for genotyping. The start and stop codons are labeled. (B) Homozygous *atvdac1* plants were complemented with the transgene (*AtVDAC1 promoter:AtVDAC1* genomic DNA). Six independent transgenic lines, wild type (WT) and the *atvdac1* mutant were analyzed by genomic PCR amplification. (C) Expression of the *AtVDAC1* gene was analyzed by RT-PCR in the six independent complemented lines, WT and the *atvdac1* mutant. The *UBQ5* transcript was amplified as an internal control.

We identified a T-DNA insertion mutant (*atvdac1*) for the *AtVDAC1* gene (At3g01280) from the Arabidopsis Biological Resource Center (ABRC), which had two T-DNAs inserted in the sixth exon and the 3′UTR of the *AtVDAC1* gene (Figure 1A). The homozygous knockout mutant was isolated and confirmed by PCR analysis (Figure 1B, the left two lines), and the expression levels of *AtVDAC1* gene were analyzed using RT-PCR (Figure 1C, the left two lines).

**Figure 2:**
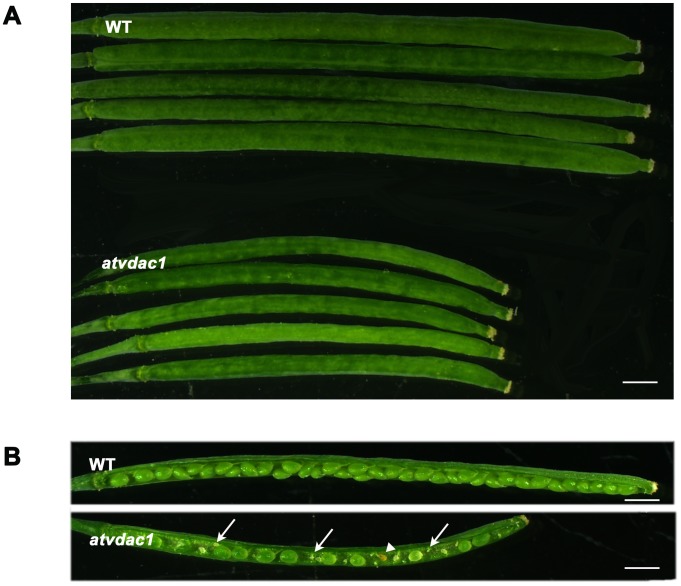
The atvdac1 mutation caused shorter siliques and reduced seed set. (A) Representative siliques from wild-type (WT) and *atvdac1* mutant plants. (B) Seed development in the representative siliques from WT and *atvdac1* mutant plants. The arrows indicate undeveloped ovules and the arrowhead indicates the aborted seed. Bars = 1 mm.

The homozygous *atvdac1* mutant had apparently shorter siliques compared with the wild type (Figure 2A, Table S1), consistent with the observations by Tateda et al. (2011) [29]. Further microscopic observation revealed that the mutant *atvdac1* had reduced seed set, that was, about 62.3% (Figure 2B, Table 1). There were two types of abnormalities in siliques from *atvdac1*: undeveloped ovules (33.4%) and aborted seeds (4.3%). As shown in Figure 2B, a plenty of undeveloped ovules were small, shrunken, and finally aborted. In contrast, full seed set was observed (Figure 2B), and the proportions of abnormalities were only about 0.8% and 0.3% respectively in wild type at the same growth condition (Table 1).

**Table 1:** Analysis of seed development in siliques from wild type (WT), *atvdac1* and reciprocal crosses between WT and *atvdac1*, showing that female fertility is affected.

Crosses (female × male)	Normal seeds (%)	Aborted seeds (%)	Undeveloped ovules (%)	Total No.
WT selfed^a^	98.9±0.2	0.3±0.1	0.8±0.2	2532
*atvdac1* selfed^a^	62.3±6.0	4.3±1.3	33.4±4.7	2583
WT × *atvdac1* b	98.0±0.5	0.6±0.6	1.4±0.3	1116
*atvdac1* × WT^b^	54.5±5.9	5.5±1.6	39.9±4.4	1057

The statistical analysis was performed in siliques from 50-day-old plants after transplantion into the soil.

a 40 siliques were examined.

b 20 siliques were examined.

**Figure 3:**
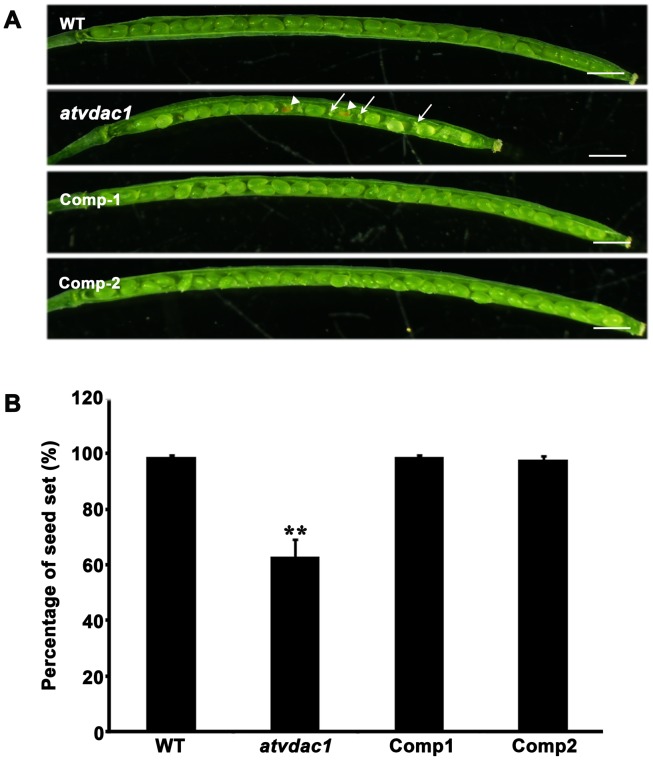
Complementation of the *atvdac1* mutant rescued the phenotype of atvdac1 plants. (A) Seed development in the representative siliques from wild-type (WT), *atvdac1* mutant and complemented *atvdac1* plants. The arrows indicate undeveloped ovules and arrowheads indicate aborted seeds. Bars = 1 mm. (B) Statistics of the seed set of complemented plants by comparison with that of *atvdac1* mutant and WT plants. The statistical analysis was performed in siliques from 50-day-old plants after transplantion into the soil. Error bars represent SD, 40 siliques were examined for each genotype. Asterisks indicate a statistically significant difference between *atvdac1* and WT (Student's *t*-test), **P<0.01.

In order to confirm that the mutation of *AtVDAC1* is responsible for the *atvdac1* phenotype, we performed a complementation experiment. The complementation construction *AtVDAC1 promoter:AtVDAC1* genomic DNA was introduced into *atvdac1* plants. Six homozygous transformants with one copy of T-DNA insertion were obtained from the transgenic *atvdac1* progeny. All these transformants were confirmed by PCR analysis using the pCAMBIA1300-specific primer PCF and *AtVDAC1* gene-specific primer PCB. The genotype analysis of wild type and the *atvdac1* mutant was also performed using primer pairs P5/LBa1, P6/LBa1 and P5/P6 as controls (Figure 1A, B). RT-PCR analysis showed that the expression of *AtVDAC1* gene could be detected in the six independent complemented lines using primer pairs P7/P8 which corresponded to the 3′UTR/5′UTR of this gene respectively (Figure 1A, C). Phenotypic analysis of two complemented lines showed that the introduced *AtVDAC1* gene rescued the defective phenotype observed in *atvdac1* plants (Figure 3A, Table S2). The percentages of seed set from the complemented plants were recovered (Figure 3B). Above results demonstrate that the defective phenotype observed in the *atvdac1* mutant results from the mutation of the *AtVDAC1* gene.

### *AtVDAC1* Is Expressed throughout Reproductive Organs

**Figure 4:**
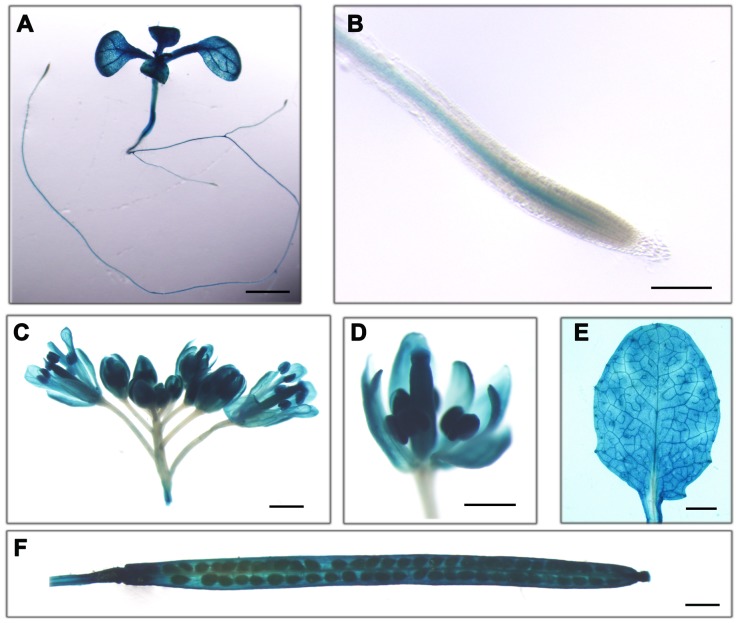
Expression pattern of AtVDAC1 in Arabidopsis, showing that GUS stains were observed in seedling (A), root tip (B), inflorescence (C), flower (D), rosette leaf (E), and silique (F) in transgenic plants carrying the *AtVDAC1 promoter:GUS* construction. Bars = 2 mm (A), 200 µm (B), 1 mm (C), 500 µm (D), 2 mm (E), and 1 mm (F).

RT-PCR and quantitative RT-PCR assays showed that *AtVDAC1* had a ubiquitous expression pattern with high transcription levels in roots, stems, leaves, inflorescences, siliques, and seedlings (data not shown). In order to investigate the *AtVDAC1* expression pattern in tissues and organs in details, *AtVDAC1 promoter:GUS* construction was introduced into wild-type plants, and histochemical GUS staining assays were performed. GUS signals were detected in the cotyledons, true leaves and roots of seedlings and rosette leaves of adult plants, basically confirming the previous results by Tateda et al. (2011) [29] and Robert et al. (2012) [30]. Especially in reproductive organs, high level of GUS activity was detected in pistils, stamens, sepals and petals including siliques where the phenotype appeared in the *atvdac1* mutant (Figure 4). This result is consistent with the analysis of microarray expression data which shows that *AtVDAC1* displays a peak of expression in reproductive organs [27].

### Mutation of *AtVDAC1* Affects Female Development

**Figure 5:**
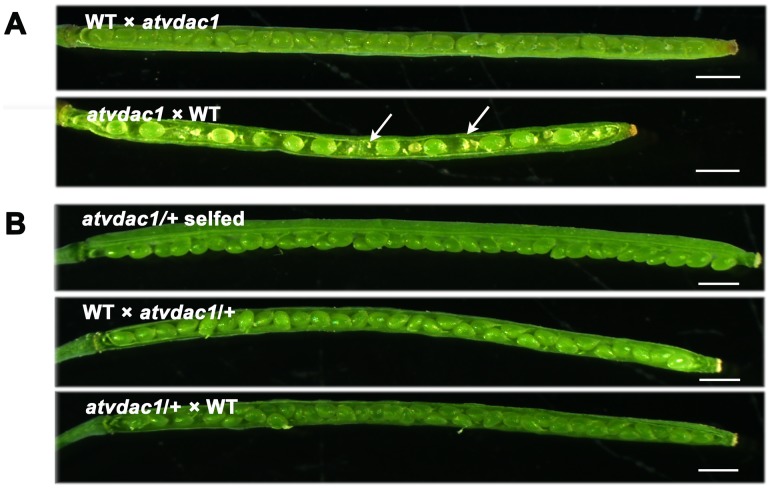
Reciprocal crosses show that female fertility was affected. (A) Seed development in representative siliques of reciprocal crosses using pollen from *atvdac1* plants to pollinate wild-type (WT) pistils and using pollen from WT plants to pollinate *atvdac1* pistils. The arrows indicate undeveloped ovules. Bars = 1 mm. (B) Seed development in the representative *atvdac1*/+ selfed silique and in representative siliques from reciprocal crosses between WT and atvdac1/+. Bars = 1 mm.

To determine whether the mutation of *AtVDAC1* affects male or female fertility, reciprocal crosses were performed between *atvdac1* and wild type. The crossed plants with *atvdac1* as the female parent and wild type as the male parent, had a similar phenotype to *atvdac1* plants. They harbored shorter siliques and showed a percentage of seed set close to that of *atvdac1* selfed plants; however, when wild type was used as the female parent in crosses with *atvdac1* plants, about 98.0% ovules produced normal seeds similar to wild-type plants (Figure 5A, Table 1, Table S1). These results indicate that the mutation of *AtVDAC1* affects female fertility.

All F1 plants from reciprocal crosses above were *atvdac1* heterozygous mutants (*atvdac1*/+) confirmed by PCR assay (data not shown), and had normal siliques and seed set (Table S3, Figure 5B). The F2 progeny of *atvdac1*/+ plants segregated approximately 3∶1 (200 wild-type phenotype plants: 66 defective phenotype plants) as a single recessive mutation. Genotype analysis using PCR assay showed that all the 66 defective phenotype plants harbored the *atvdac1* homozygous mutation (*atvdac1*/*atvdac1*), and there were 136 *atvdac1* heterozygous (*atvdac1*/+) plants and 64 wild-type (+/+) plants in the 200 wild-type phenotype plants (Table 2). This result indicates the linkage between the phenotype and T-DNA insertion in the *AtVDAC1* gene. Because the progeny of *atvdac1*/+ exhibited the Mendelian 1∶2∶1 (66∶136∶64) segregation pattern, the *atvdac1* is a sporophytic mutant. In addition, reciprocal crosses between *atvdac1*/+ plants and wild-type (+/+) plants were also performed. As shown in figure 5B and Table S3, the crossed siliques had normal length and seed set. F1 plants from both crosses showed the same segregation ratio of nearly 1∶1 (with T-DNA insertion to without T-DNA insertion = 176∶192 and 239∶221, respectively) (Table 2), which means female and male transmissions of the *atvdac1* allele from heterozygotes were not reduced. Therefore, the mutation of *AtVDAC1* does not affect either male or female gametophytic development.

**Table 2:** The segregation ratio of progeny from *atvdac1*/+ selfed and reciprocal crosses between wild type (WT) and *atvdac1*/+.

Crosses (female × male)	*atvdac1*/*atvdac1* with T-DNA insertion (W1)	*atvdac1*/+ with T-DNA insertion (W2)	+/+ without T-DNA insertion (WO)	Ratio (W1+W2):WO	TE_F_	TE_M_
*atvdac1*/+ selfed^a^	66	136	64	3.16∶1	NA	NA
WT *× atvdac1/+* b	0	176	192	0.92∶1	NA	92%
*atvdac1*/+ × WT^b^	0	239	221	1.08∶1	100%	NA

TE, transmission efficiency  =  (W1+W2)/WO ×100%; TE_F_, female transmission efficiency.

TE_M_, male transmission efficiency; NA, not applicable.

a The ratio was calculated using seeds from ten *atvdac1*/+ plants.

b The ratio was calculated using seeds from eight plants.

**Figure 6:**
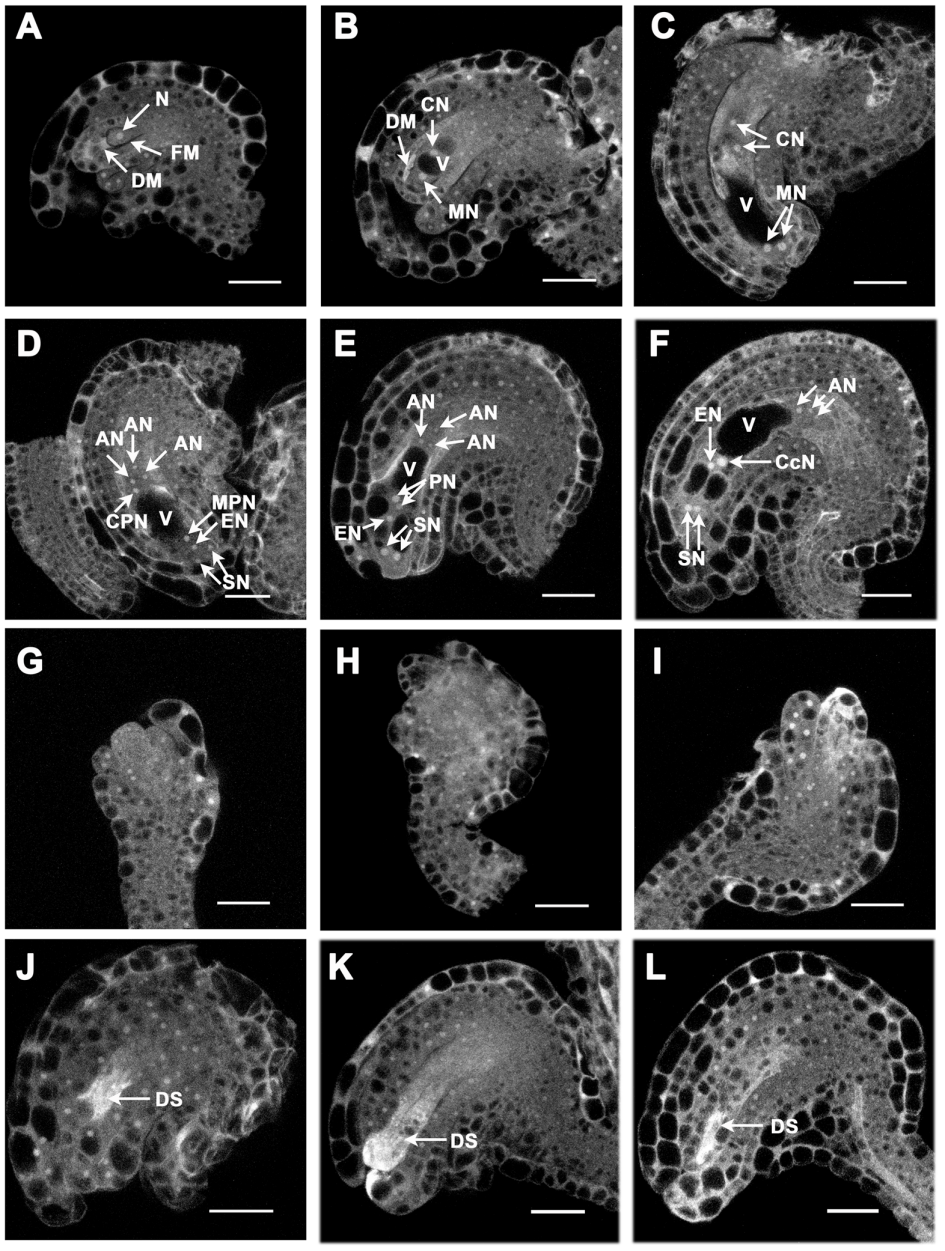
Ovule development in wild-type (WT) and *atvdac1* siliques revealed by confocal laser scanning microscopy (CLSM). (A–F) Female gametogenesis in WT siliques at stage FG1 (A), FG3 (B), FG4 (C), early FG5 (D), late FG5 (E), FG6 (F). (G–L) Abnormal ovules in *atvdac1* siliques at stage FG1 (G), FG3 (H), FG4 (I), FG5 (J, K), FG6 (L). Abbreviations: AN, antipodal nucleus (nuclei); CN, chalazal nucleus (nuclei); CcN, central cell nucleus; CPN, chalazal polar nucleus; DM, degenerated megaspores; DS, degenerated structure; EN, egg cell nucleus; FM, functional megaspore; MN, micropylar nucleus (nuclei); MPN, micropylar polar nucleus; N, nucleus; PN, polar nuclei; SN, synergid nuclei; V, vacuole. Bars = 20 µm.

To further investigate the phenotype of undeveloped ovules observed in *atvdac1* siliques, ovule development in wild-type and mutant plants was examined via confocal laser scanning microscopy (CLSM) [34]. In wild-type plants, a single hypodermal archesporial cell directly differentiates into a megaspore mother cell that undergoes meiosis to generate four haploid megaspores (stage FG0). As shown in Figure 6A, the chalazal-most megaspore survived, enlarged and became the functional megaspore (FM); and the micropylar megaspores degenerated (DM), as shown by their strong autofluorescence in wild type (stage FG1). The haploid functional megaspore underwent three rounds of mitosis with cellularization and fusion of polar nuclei (stages FG3–FG6, Figure 6B-F). But in *atvdac1* pistils, we observed smaller ovules that lacked the embryo sac structure with one functional megaspore when other normal ones finished sporogenesis and reached the FG1 stage (Figure 6G). This result suggests that the process of sporogenesis was affected so the uninucleate female gametophyte could not form in abnormal ovules. During FG3-FG6 stages, abnormal ovules without nuclei and embryo sac structures or with degraded structures (DS) shown as strong autofluorescence, were also observed (Figure 6H-L).

### Mutation of *AtVDAC1* Affects ΔΨ and ATP Synthesis

**Figure 7:**
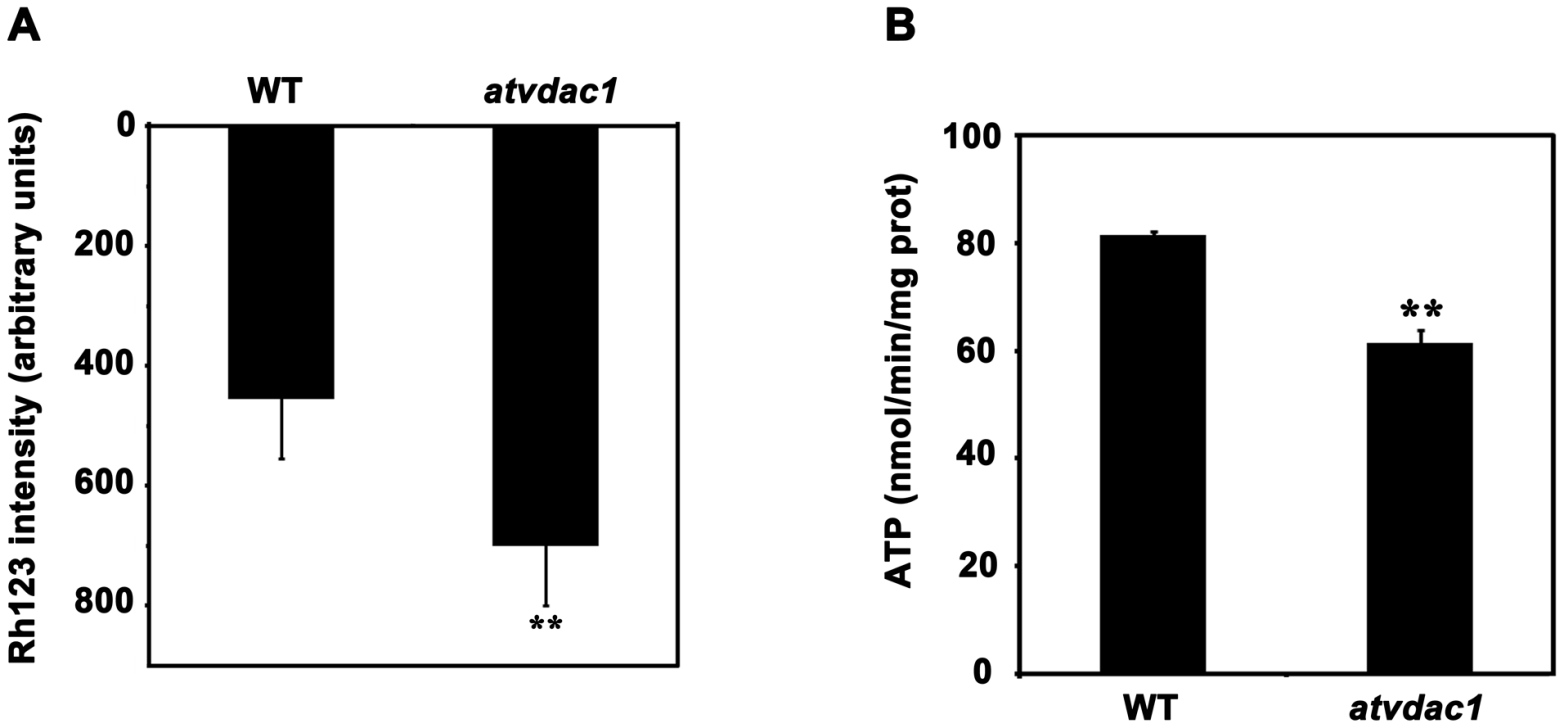
Mitochondrial transmembrane potential (ΔΨ) and ATP synthesis rate were lower in *atvdac1* than those in wild type (WT). (A) Isolated mitochondria (1 mg/mL) prepared from WT and *atvdac1* mutant plants were treated with Rhodamine123 (Rh123) for 20 min, and ΔΨ was measured by Rh123 uptake. (B) ATP synthesis rate was measured in mitochondria prepared from WT and *atvdac1* mutant plants. Error bars represent SD (n≥3). Asterisks indicate statistically significant differences between *atvdac1* and WT (Student's *t*-test), **P<0.01.

In order to find out whether AtVDAC1 is involved in regulation of the mitochondrial transmembrane potential (ΔΨ) in *Arabidopsis*, we isolated mitochondria from wild-type and *atvdac1* plants respectively, and measured the ΔΨ using Rhodamine123. As shown in Figure 7A, the ΔΨ in *atvdac1* was obviously lower than that in wild type. Mitochondria are the energy powerhouse in eukaryotic cells, so oxidative phosphorylation efficiency may be affected as a result of abnormal functional state of mitochondria. ATP synthesis rate was then measured using isolated mitochondria. The mitochondria from *atvdac1* plants produced approximately 25% less ATP than that from wild-type plants (Figure 7B). These results indicate that the mutation of *AtVDAC1* affects ΔΨ and ATP synthesis of mitochondria.

## Discussion

VDACs play crucial roles in regulating metabolic and energetic functions of mitochondria [4], [5], [8], [35] and are involved in mitochondria-mediated apoptosis in mammalian cells [10], [36]. But the knowledge on plant VDACs is still limited. As to the function of AtVDAC1 on reproductive development, one group reported that the pollen development was affected in *vdac1* plants [29]. The other group suggested that the decreased seed production resulted from zygotic or early embryo lethality [30]. In this work, we found that there were plenty of undeveloped ovules in *atvdac1* siliques which mainly resulted from the defective female development. Results indicated that mitochondrial transmembrane potential (ΔΨ) and ATP synthesis rate were reduced in the mitochondria isolated from *atvdac1* plants.

We analyzed the T-DNA insertion mutant *atvdac1* (SALK_034653) for the *AtVDAC1* gene (At3g01280). In *atvdac1* siliques, approximately 37.7% of ovules did not develop into mature seeds (Table 1). Only the homozygous *atvdac1* mutant (*atvdac1*/*atvdac1*) had the defective phenotype (Figure 2) and the *atvdac1* heterozygous mutant (*atvdac1*/+) had full seed set (Figure 5B), so the reduced seed set did not result from zygotic or embryo lethality. Genetic analyses were then used to investigate the reason of the reduced seed set. Firstly, reciprocal crosses between homozygous *atvdac1* and wild type were performed according to the approach described by Motamayor et al. (2000) [37] and Wang et al. (2012) [38]. When wild-type pistils were pollinated by mutant pollen, the crossed siliques had normal seed set; however, *atvdac1* siliques showed the same defective phenotype regardless of the pollen was from wild-type or mutant plants (Figure 2B, 5A; Table 1), indicating that female fertility in *atvdac1* plants was affected.

Sporophytic mutations affect the diploid sporophyte generation of the life cycle of plants and exhibit Mendelian 1∶2∶1 segregation patterns; on the contrary, gametophytic mutations affect the gametophyte generation and exhibit altered segregation patterns [39], [40]. The progeny of *atvdac1*/+ exhibited the Mendelian 1∶2∶1 segregation pattern (Table 2). The results from reciprocal crosses between *atvdac1*/+ plants and wild-type plants indicated that female and male transmissions of the *atvdac1* allele from heterozygotes were not affected (Table 2). Accordingly, the mutation of *AtVDAC1* does not affect either male or female gametophytic development and is a sporophytic mutation. Interestingly abnormal ovules were observed using a confocal laser scanning microscope in stage FG1 (Figure 6G), pointing to the earlier defect in the process of megasporogenesis. As ATP synthesis rate was reduced in the mitochondria isolated from *atvdac1* plants (Figure 7B), we hypothesize that the process of meiosis might be affected during the stage of megasporogenesis. Many motor proteins driven by ATP hydrolysis are involved in meiosis; therefore, enough amount of ATP is needed in this process [41], [42]. In *Arabidopsis*, many sporophytic mutants affecting sporogenesis have been reported [37], [43]–[46]. Moreover, abnormal ovules without nuclei and embryo sac structures or with degraded structures (DS) were also observed at stages FG3-FG6 (Figure 6H-L). We suggest that the defects might directly result from the abnormal megasporogenesis or from the affected sporophytic tissues. Previous studies demonstrated that integuments in sporophytic tissues played important roles on the control of megagametogenesis [47]–[49].

Several lines of evidence have been shown that mitochondria are deeply involved in male gametophytic development and many mutations of mitochondrial genes affect male gametophytic development, even resulting in cytoplasmic male sterility (CMS) [50], [51]. In our study, only female development was affected by genetic analyses (Table 1, 2) in *atvdac1* plants, which may be result from the sporophytic mutation of *atvdac1*. The female gametocyte is included in the diploid sporophytic tissues; therefore, female development may be affected by the defect of sporophytic tissues. Till now, we cannot exclude that the microsporogenesis is affected in the *atvdac1* mutant only according to the results from genetic analyses. Future investigations on the microsporogenesis will provide further information for this issue.

Interestingly, in the study of Tateda and coworkers, neither *vdac1-3* (SALK_039833) nor *vdac1-5* (SALK_058473C) homozygous mutant was identified in the progeny of self-crossed heterozygous plants [29]. The positions of T-DNA insertions within the 5′ region of the *AtVDAC1* gene may result in complete disruption of this gene in *vdac1-3* and *vdac1-5* plants, implying that AtVDAC1 plays important roles in the plant growth and development. In our study, the *atvdac1* (SALK_034653) mutant harbored two T-DNAs inserted in the 3′ end (the last exon and 3′UTR) of the *AtVDAC1* gene (Figure 1A). The *AtVDAC1* transcripts including first five exons were detected in homozygous *atvdac1* plants by RT-PCR analysis (Figure S1), implying that the *atvdac1* plants might accumulate truncated AtVDAC1 polypeptides retaining partial activity. Therefore, the homozygous *atvdac1* plants could be obtained but the female development was affected, different from the *vdac1-6* (SALK_011520C) mutant with a T-DNA in the fifth intron, exhibiting defective pollen development [29]. Different phenotypes might result from the different positions of T-DNA insertions. This phenomenon that different mutations in the same gene exhibit different phenotypes, was also observed in other genes such as *SWI1* [52].

Several studies have been performed to determine the localization of AtVDACs using various experimental methods. The mitochondrial localization of AtVDACs was observed in the protoplasts of AtVDACs-GFP transgenic plants and in onion epidermal cells using AtVDACs–GFP fusion constructs [22], [29]. However, AtVDAC3 was also identified in plasma membrane fractions of *Arabidopsis* cell suspensions [53]. Recently, biochemical evidence has revealed that AtVDAC1, AtVDAC2 and AtVDAC3 were targeted to both mitochondria and plasma membrane in differential abundance but their functions seemed to be mainly associated with mitochondria [30]. In plants, evidence regarding the role of VDACs in mitochondrial function is mainly based on their mitochondrial localization [28]. Several lines of evidence show that the Bcl-2 family proteins could interact with VDAC to regulate the mitochondrial transmembrane potential (ΔΨ) during mitochondria-mediated apoptosis in mammalian cells [10], [11], [54]. Several lines of evidence indicate cytological and biochemical similarities between plant and mammalian apoptosis [55]. In order to investigate the relationship between plant VDAC and the ΔΨ, we measured ΔΨ in isolated mitochondria prepared from wild-type and *atvdac1* mutant plants. Our result indicated that the mutation of *AtVDAC1* resulted in the decline of ΔΨ (Figure 7A), similar as that detected in protoplasts [29]. Therefore we suppose that AtVDAC1 plays an important role in maintaining the steady state of ΔΨ in *Arabidopsis*. That VDACs are involved in mitochondrial respiration in other species have been reported [15],[56],[57]. In human cells, diminished VDAC1 expression resulted in decreased ATP synthesis and cytosolic ADP and ATP levels [58]. In *Arabidopsis*, we previously reported that AtVDAC3 was involved in regulating respiration pathways and ATP levels during seed germination at low temperature [59]. In *atvdac1* mutant, ATP synthesis rate was also obviously reduced in isolated mitochondria (Figure 7B). Genetic characterization of mutants indicates that mitochondria-associated proteins play important roles in female development in *Arabidopsis* [60]–[63]. For example, a mutation of *SDH1-1* encoding the flavoprotein subunit of mitochondrial complex II (succinate dehydrogenase [SDH]) affects the embryo sac development [62]. The decline of ΔΨ and ATP synthesis means the abnormal function state of mitochondria and reduced energy supply. Shortage of energy supply could compel plants to finish the reproductive process as soon as possible. As to a silique, shortage ATP supply would not meet the need of the whole ovules development, so reduced seed set could be observed.

## Materials and Methods

### Plant Material and Growth Conditions

All *Arabidopsis thaliana* plants used in this study were derived from the Columbia ecotype background (Col-0). Seeds from *atvdac1* (SALK_034653) were obtained from the Arabidopsis Biological Resource Center (http://abrc.osu.edu/) [30]. The genotype of the T-DNA insertion line for *AtVDAC1* (At3g01280) was confirmed by PCR analysis with primers: P5, P6 and LBa1. Seeds were surface-sterilized with chloride gas. After cold treatment at 4°C for 2 days, seeds were sown on the medium containing 0.5 × MS salts (MS, Sigma-Aldrich, St. Louis, MO, USA), 1% sucrose and 0.8% agar which was supplemented with 15 mg/L hygromycin as required. 7-day-old seedlings were transferred to soil and grown at 22°C under a 16-hr-light/8-hr-dark cycle.

### *AtVDAC1* Complementation Experiment

The genomic DNA fragment of *AtVDAC1* (5′UTR, exons, introns and 3′UTR included) and the 1.8 kb promoter were amplified using *Arabidopsis* genomic DNA as templates with primer pairs as follows: for the promoter using V1PF and V1PBG, for the genomic DNA fragment using V1GF and V1GB. The two fragments were cloned into the pCAMBIA1300 vector (harboring the resistant gene to hygromycin) by the sites of *Sac*I/*Bam*HI and *Bam*HI/*Xba*I to generate *AtVDAC1 promoter:AtVDAC1* genomic DNA construction which then was introduced into *atvdac1* plants according to the method of Clough and Bent (1998) [64]. The transformants were selected on 15 mg/L hygromycin and further verified by PCR assays using primer pairs PCF and PCB. *UBQ5* (At3g62250) was amplified using primer pairs UBQF and UBQB in RT-PCR analyses.

### Construction of *AtVDAC1 Promoter:GUS* and Histochemical GUS Staining Assays

The *AtVDAC1* promoter (1953 bp upstream from the start codon) was amplified from *Arabidopsis* genomic DNA with the primer pairs, V1PFP and V1PBC. The *AtVDAC1* promoter fragment was then cloned into pCAMBIA1300221-GUS vector (harboring the resistant gene to hygromycin) by the *Pst*I/*Bam*HI sites to generate *AtVDAC1 promoter:GUS* construction.

Plant tissue samples were prefixed with cold 90% acetone for 15 min, and then washed twice with sterilized water. Samples were incubated in the GUS staining solution (1 mg/mL 5-bromo-4-chloro-3-indolyl-β-D-glucuronide, 2 mM potassium ferricyanide and potassium ferrocyanide, 10 mM EDTA and 0.1% Triton X-100 in 50 mM sodium phosphate buffer, pH 7.0) at 37°C for 6–8 hr after vacuum-infiltrated for 10 min. Stained samples were cleared in 75% ethanol solution and 25% acetic acid solution, and afterward observed with a Olympus SZX16 microscope (Olympus, Tokyo, Japan).

### Confocal Laser Scanning Microscopic (CLSM) Observation

The CLSM observation of ovules was performed as described by Christensen et al. (1997) [34] and Shi et al. (2005) [65] with slight modifications. The pistils were fixed in 4% glutaraldehyde (in 12.5 mM cacodylate, pH 6.9) and kept under a vacuum for 2 hr. After further fixation overnight at room temperature, tissues were dehydrated through an ethanol series. Then the tissues were cleared in 2∶1 (v/v) benzyl benzoate:benzyl alcohol for at least 1 hr. The pistils were dissected, sealed under cover slips and then observed with a Zeiss LSM510 META confocal laser scanning microscope (Zeiss, Jena, Germany).

### Isolation of Mitochondria

The rosette leaves of plants grown in a greenhouse for 4 weeks were used as starting materials. Isolation of mitochondria was performed as described by Day and Hanson (1978) [66] with following modifications. The materials were ground in an ice-cold mortar with extraction buffer (0.3 M sucrose, 1 mM EDTA, 1 mg/mL bovine serum albumin (BSA), 0.6% [w/v] polyvinylpyrrolidone 40, 0.5 mM dithiothreitol (DTT), 0.2 mM phenylmethylsulfonyl fluoride (PMSF), and 20 mM This-HCl, pH 8.0). The homogenate was filtered through 4 layers of gauze and centrifuged at 3,000 g for 10 min. The supernatant was centrifuged at 11,000 g for 20 min to collect the pellet. The pellet was resuspended in extraction buffer and subjected to low-speed (1,500 g) and high-speed (11,000 g) centrifugations. The pellet was resuspended in a small volume of extraction buffer and loaded on top of a sucrose “cushion” (0.6 M sucrose, 1 mM EDTA, 1 mg/mL BSA, 0.5 mM DTT, 0.2 mM PMSF, and 20 mM This-HCl, pH 8.0). The cushion was centrifuged at 11,000 g for 20 min. The pellet was washed twice in resuspension buffer (0.3 M Sucrose, 1 mg/mL BSA, 20 mM This-HCl, pH 8.0).

### Measurement of ΔΨ and ATP synthesis rate in isolated mitochondria

Measurement of mitochondrial transmembrane potential (ΔΨ) in isolated mitochondria was performed with the method described by Narita et al. (1998) [67]. ΔΨ was assessed by measuring the ΔΨ-dependent uptake of Rhodamine123 (Rh123) by using a spectrophotometer (Hitachi F-4500, Tokyo, Japan) with excitation at 505 nm and emission at 534 nm after addition of 10 µM Rh123 to a mitochondrial suspension. Measurement of ATP synthesis rate in isolated mitochondria was performed according to the method of Meyer et al. (2009) [68] using the ATP Bioluminescent Assay Kit (Sigma-Aldrich, St. Louis, MO, USA). Mitochondria were incubated in respiration buffer containing 10 mM Glu, 10 mM malate, 12 µM CoA, 0.2 mM TPP, 2 mM NAD^+^, and 1 mM ADP for exactly 10 min. Then, TCA was added to a final concentration of 2.3% (w/v). After centrifugation for 15 min at 20,000 g, the supernatant was diluted for 100 times using 25 mM Tricine buffer (pH 7.8) and then used for ATP production estimation.

## Supporting Information

Figure S1 **RT-PCR analysis of** ***AtVDAC1*** **gene transcripts in wild type (WT) and the** ***atvdac1*** **mutant.** (A) Schematic diagram of the *AtVDAC1* gene structure and the T-DNA insertion sites in the *atvdac1* mutant. Closed boxes indicate exons, and arrowheads indicate the positions of primers. The start and stop codons are labeled. (B) RT-PCR analysis of *AtVDAC1* gene transcripts in WT and *atvdac1* mutant. The *UBQ5* transcript was amplified as an internal control.
(TIF)

Table S1 **Statistics for lengths of siliques from wild type (WT),** ***atvdac1*** **and reciprocal crosses between wild type (WT) and** ***atvdac1*** **.**
(DOCX)

Table S2 **Statistics for lengths of siliques from wild type (WT),** ***atvdac1*** **and complemented lines.**
(DOCX)

Table S3 **Statistics for lengths of siliques from** ***atvdac1*** **/+ selfed and reciprocal crosses between wild type (WT) and** ***atvdac1*** **/+.**
(DOCX)

Table S4 **Sequences of the primers used in this study.**
(DOCX)
