# Supplementary figures and images for: Arabidopsis Voltage-Dependent Anion Channel 1 (AtVDAC1) Is Required for Female Development and Maintenance of Mitochondrial Functions Related to Energy-Transaction
Source: PLoS One. 2014 Sep 5;9(9):e106941. doi: 10.1371/journal.pone.0106941 (PMC4156401; doi:10.1371/journal.pone.0106941)

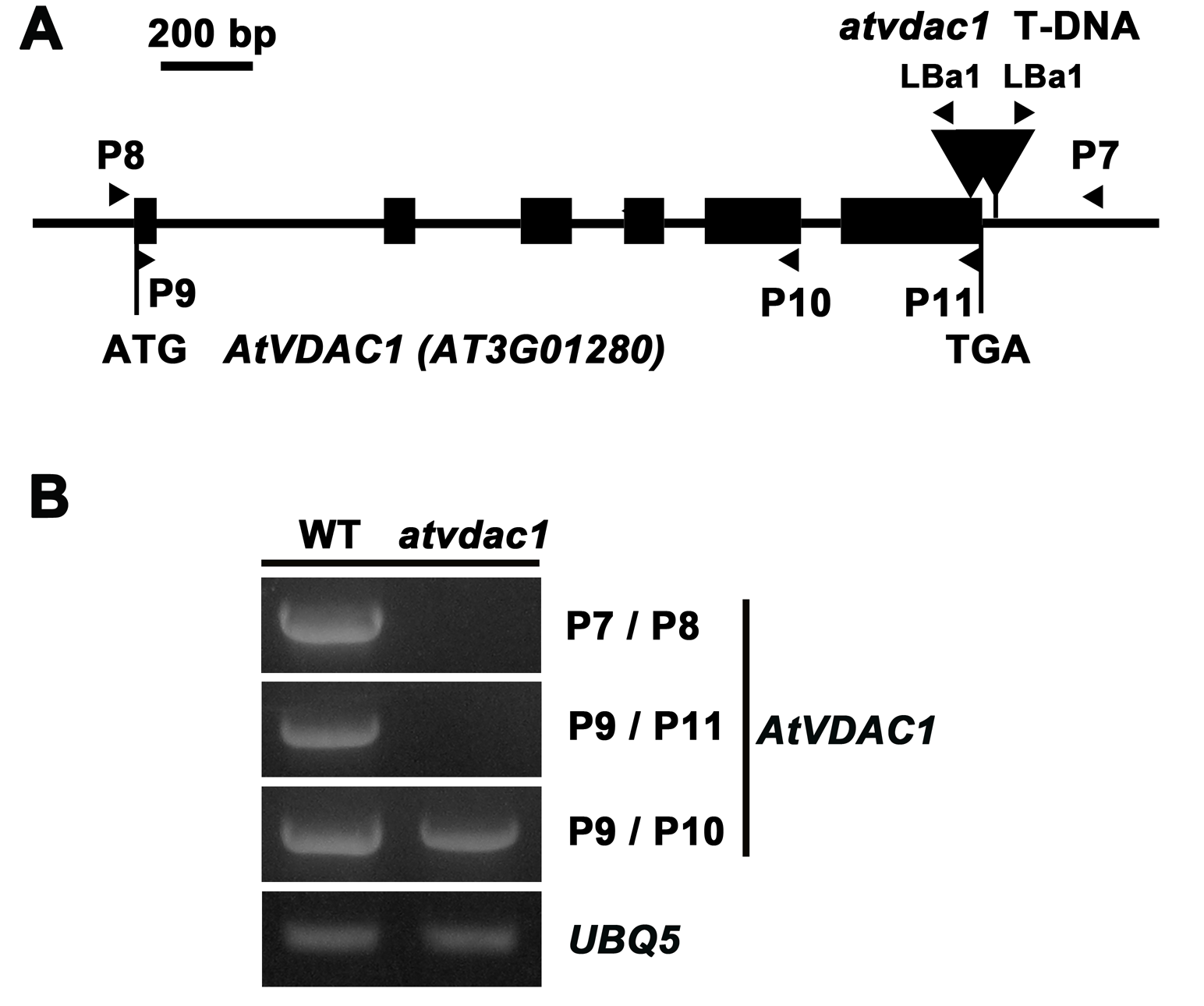

Supplement: Figure S1 — RT-PCR analysis of AtVDAC1 gene transcripts in wild type (WT) and the atvdac1 mutant. (A) Schematic diagram of the AtVDAC1 gene structure and the T-DNA insertion sites in the atvdac1 mutant. Closed boxes indicate exons, and arrowheads indicate the positions of primers. The start and stop codons are labeled. (B) RT-PCR analysis of AtVDAC1 gene transcripts in WT and atvdac1 mutant. The UBQ5 transcript was amplified as an internal control. (TIF) [file pone.0106941.s001.tif]
