# Supplementary material for: Arabidopsis Voltage-Dependent Anion Channel 1 (AtVDAC1) Is Required for Female Development and Maintenance of Mitochondrial Functions Related to Energy-Transaction
Source: PLoS One. 2014 Sep 5;9(9):e106941. doi: 10.1371/journal.pone.0106941 (PMC4156401; doi:10.1371/journal.pone.0106941)
Supplement: Table S1 — Statistics for lengths of siliques from wild type (WT), atvdac1 and reciprocal crosses between wild type (WT) and atvdac1 . (DOCX) [file pone.0106941.s002.docx]

**Table S1.** Statistics for lengths of siliques from wild type (WT), *at*vdac1 and reciprocal crosses between wild type (WT) and *at*vdac1.

| Crosses (female × male) | Silique length (cm) |
| --- | --- |
| WT selfed^a^ | 1.51±0.03 |
| *atvdac1* selfed^a^ | 1.10±0.17 |
| WT × *atvdac1*^b^ | 1.42±0.06 |
| *atvdac1* × WT ^b^ | 0.98±0.11 |

The statistical analysis was performed in siliques from 50-day-old plants after transplantion into the soil.

^a^, 40 siliques were examined.

^b^, 20 siliques were examined.
