# Supplementary material for: Arabidopsis Voltage-Dependent Anion Channel 1 (AtVDAC1) Is Required for Female Development and Maintenance of Mitochondrial Functions Related to Energy-Transaction
Source: PLoS One. 2014 Sep 5;9(9):e106941. doi: 10.1371/journal.pone.0106941 (PMC4156401; doi:10.1371/journal.pone.0106941)
Supplement: Table S2 — Statistics for lengths of siliques from wild type (WT), atvdac1 and complemented lines. (DOCX) [file pone.0106941.s003.docx]

**Table S2.** Statistics for lengths of siliques from wild type (WT), *at*vdac1 and complemented lines.

| Plants | Silique length (cm) |
| --- | --- |
|  |  |
| WT^a^ | 1.51±0.02 |
| *atvdac1*^a^ | 1.13±0.11 |
| Comp-1^a^ | 1.52±0.06 |
| Comp-2^a^ | 1.52±0.04 |

The statistical analysis was performed in siliques from 50-day-old plants after transplantion into the soil.

^a^, 40 siliques were examined.
