# Supplementary material for: Arabidopsis Voltage-Dependent Anion Channel 1 (AtVDAC1) Is Required for Female Development and Maintenance of Mitochondrial Functions Related to Energy-Transaction
Source: PLoS One. 2014 Sep 5;9(9):e106941. doi: 10.1371/journal.pone.0106941 (PMC4156401; doi:10.1371/journal.pone.0106941)
Supplement: Table S3 — Statistics for lengths of siliques from atvdac1 /+ selfed and reciprocal crosses between wild type (WT) and atvdac1 /+. (DOCX) [file pone.0106941.s004.docx]

**Table S3.** Statistics for lengths of siliques from *at*vdac1/+ selfed and reciprocal crosses between wild type (WT) and *at*vdac1/+.

| Crosses (female × male) | Silique length (cm) |
| --- | --- |
| *atvdac1*/+ selfed^a^ | 1.53±0.04 |
| WT × *atvdac1*/+ ^b^ | 1.40±0.04 |
| *atvdac1*/+ × WT^b^ | 1.41±0.06 |

The statistical analysis was performed in siliques from 50-day-old plants after transplantion into the soil.

^a^, 40 siliques were examined.

^b^, 20 siliques were examined.
