# Supplementary material for: Arabidopsis Voltage-Dependent Anion Channel 1 (AtVDAC1) Is Required for Female Development and Maintenance of Mitochondrial Functions Related to Energy-Transaction
Source: PLoS One. 2014 Sep 5;9(9):e106941. doi: 10.1371/journal.pone.0106941 (PMC4156401; doi:10.1371/journal.pone.0106941)
Supplement: Table S4 — Sequences of the primers used in this study. (DOCX) [file pone.0106941.s005.docx]

**Table S4.** Sequences of the primers used in this study.

| Primers | Sequences (5' to 3') |
| --- | --- |
| P5 | TTATTACAGGCCAACAATGCC |
| P6 | GTGATTGGCTCCAATGTCTTG |
| P7 | CTGAATATGCAATTTTCATTATGACAC |
| P8 | CCTCCAACTTTCTCAGATAAGCAAC |
| P9 | ATGGTGAAAGGTCCCGGTC |
| P10 | ACAGTAAGGGAGGCAATTAAGTCC |
| P11 | TCAAGGCTTGAGTGCGAGAG |
| LBa1 | TGGTTCACGTAGTGGGCCATCG |
| V1PF | GAGCTCTGATACCAGCACCATATGCG |
| V1PBG | GGATCCAAGAGTTTTCCTGTTTATC |
| V1GF | GGATCCCTTTCTCTGATAATATTCG |
| V1GB | TCTAGACTGAATATGCAATTTTCATTATG |
| PCF | GAACGATAGCCTTTCCTTTA |
| PCB | TCTTCAGCAACTCGACTGAC |
| UBQF | GGTGCTAAGAAGAGGAAGAA |
| UBQB | CTCCTTCTTTCTGGTAAACGT |
| V1PFP | CTGCAGTGATACCAGCACCATATGCG |
| V1PBC | GGATCCTGTTGCTTATCTGAGAAAG |
